# Comparison of an Artificial Intelligence–Enabled Patient Decision Aid vs Educational Material on Decision Quality, Shared Decision-Making, Patient Experience, and Functional Outcomes in Adults With Knee Osteoarthritis: A Randomized Clinical Trial

**DOI:** 10.1001/jamanetworkopen.2020.37107

**Published:** 2021-02-18

**Authors:** Prakash Jayakumar, Meredith G. Moore, Kenneth A. Furlough, Lauren M. Uhler, John P. Andrawis, Karl M. Koenig, Nazan Aksan, Paul J. Rathouz, Kevin J. Bozic

**Affiliations:** 1 Dell Medical School at the University of Texas at Austin, Austin; 2 University of Cincinnati College of Medicine, Cincinnati, Ohio; 3 Chicago Medical School, North Chicago, Illinois; 4 Harbor-UCLA Medical Center, West Carson, California

## Abstract

**Importance:**

Decision aids can help inform appropriate selection of total knee replacement (TKR) for advanced knee osteoarthritis (OA). However, few decision aids combine patient education, preference assessment, and artificial intelligence (AI) using patient-reported outcome measurement data to generate personalized estimations of outcomes to augment shared decision-making (SDM).

**Objective:**

To assess the effect of an AI-enabled patient decision aid that includes education, preference assessment, and personalized outcome estimations (using patient-reported outcome measurements) on decision quality, patient experience, functional outcomes, and process-level outcomes among individuals with advanced knee OA considering TKR in comparison with education only.

**Design, Setting, and Participants:**

This randomized clinical trial at a single US academic orthopedic practice included 129 new adult patients presenting for OA-related knee pain from March 2019 to January 2020. Data were analyzed from April to May 2020.

**Intervention:**

Patients were randomized into a group that received a decision aid including patient education, preference assessment, and personalized outcome estimations (intervention group) or a group receiving educational material only (control group) alongside usual care.

**Main Outcomes and Measures:**

The primary outcome was decision quality, measured using the Knee OA Decision Quality Instrument (K-DQI). Secondary outcomes were collaborative decision-making (assessed using the CollaboRATE survey), patient satisfaction with consultation (using a numerical rating scale), Knee Injury and Osteoarthritis Outcome Score Joint Replacement (KOOS JR) score, consultation time, TKR rate, and treatment concordance.

**Results:**

A total of 69 patients in the intervention group (46 [67%] women) and 60 patients in the control group (37 [62%] women) were included in the analysis. The intervention group showed better decisional quality (K-DQI mean difference, 20.0%; SE, 3.02; 95% CI, 14.2%-26.1%; *P* < .001), collaborative decision-making (CollaboRATE, 8 of 69 [12%] vs 28 of 60 [47%] patients below median; *P* < .001), satisfaction (numerical rating scale, 9 of 65 [14%] vs 19 of 58 [33%] patients below median; *P* = .01), and improved functional outcomes at 4 to 6 months (mean [SE] KOOS JR, 4.9 [2.24] points higher in intervention group; 95% CI, 0.8-9.0 points; *P* = .02). The intervention did not significantly affect consultation time (mean [SE] difference, 2.23 [2.18] minutes; *P* = .31), TKR rates (16 of 69 [23%] vs 7 of 60 [12%] patients; *P* = .11), or treatment concordance (58 of 69 [84%] vs 44 of 60 [73%] patients; *P* = .19).

**Conclusions and Relevance:**

In this randomized clinical trial, an AI-enabled decision aid significantly improved decision quality, level of SDM, satisfaction, and physical limitations without significantly impacting consultation times, TKR rates, or treatment concordance in patients with knee OA considering TKR. Decision aids using a personalized, data-driven approach can enhance SDM in the management of knee OA.

**Trial Registration:**

ClinicalTrials.gov Identifier: NCT03956004

## Key Points

**Question:**

How does an artificial intelligence (AI)–enabled decision aid generated using patient-reported outcome measurements compare with education only on decision quality, patient satisfaction, and functional outcomes among individuals with knee osteoarthritis considering total knee replacement?

**Findings:**

This randomized clinical trial of 129 patients demonstrated statistically significant improvement in decision quality, level of shared decision-making, patient satisfaction, and functional outcomes in patients using an AI-enabled decision aid.

**Meaning:**

These findings suggest that AI-enabled decision aids incorporating patient-reported outcome measurement data provide a personalized, data-driven approach to shared decision-making for the surgical management of knee osteoarthritis.

## Teaser

This randomized clinical trial of patients with knee osteoarthritis assessed the effect of an artificial intelligence–enabled decision aid on decision quality, patient satisfaction, and functional outcomes when compared with patient education only.

## Introduction

Osteoarthritis (OA) of the knee has increased in prevalence and now represents a major public health concern and driver of health care spending.^1,2,3,4,5^ Treatments for knee OA range from activity modification, weight loss, physical therapy, and oral analgesics to joint injections and joint replacement surgery.^6^ The presence of multiple treatment options highlights the preference-sensitive nature of OA management and an opportunity for shared decision-making (SDM).^7,8^ SDM is a concept that integrates effective communication and clinician-patient relationship building to understand patient preferences, values, and needs, with the transfer of knowledge regarding treatments, risks, benefits, and alternatives prior to making informed decisions.^9,10^ Simultaneously, there is growing interest in incorporating patient-reported outcome measurements (PROMs) in the decision-making process.^11,12^ PROMs quantify physical, emotional, and social aspects of health from the patient’s perspective.^13,14^ These tools have revolutionized patient outcomes research and are increasingly used at the point-of-care for clinical decision support.^9,15,16,17^

Baseline PROM scores can estimate postoperative outcomes when measured against scoring thresholds indicating whether patients are more or less likely to experience clinically meaningful improvement after total knee replacement (TKR)—a procedure consistently providing pain relief, functional restoration, and quality of life (QoL) improvement for advanced OA.^14,18^ This function of PROMs can be augmented by artificial intelligence (AI) and machine learning to synthesize complex relationships within large data sets.^19,20,21^ Combining the analytical power of machine learning with clinical and patient-generated data can provide personalized estimations of health outcomes and minimize guesswork during decision-making. We sought to evaluate an AI-enabled patient decision aid (Joint Insights, OM1) delivering patient education, an interactive preferences assessment, and personalized outcome reports generated by a machine learning algorithm using a large national data set.

The primary objective of this study was to evaluate how an AI-enabled decision aid (intervention group) affected decision quality for patients with knee OA considering TKR compared with the provision of digital patient education and usual care alone (control group). Secondarily, this study quantified differences between intervention and control groups on the patient’s perspective of SDM during the clinical encounter, consultation satisfaction, change in functional outcome, consultation duration, TKR rates, and treatment concordance.

## Methods

### Trial Design

We performed a parallel randomized clinical trial with a 1:1 allocation between cohorts at a musculoskeletal integrated practice unit (IPU) in an academic center in the US serving a diverse population. Two orthopedic surgeons work with a coordinated multidisciplinary team—including an advanced practice health professional, physical therapist, behavioral therapy–trained social worker, and nutritionist—in a collocated outpatient facility. Services include structured exercise programs, imaging, joint injections, weight loss counseling, dietary advice, social support, smoking and alcohol cessation, behavioral therapies, pain management, and surgery where appropriate.^22^ PROMs are collected prior to or on arrival in clinic and at follow-up time points as a standard of care.^23^ The study was reviewed and approved by the University of Texas at Austin, Dell Medical School institutional review board, and verbal informed consent was obtained from participants. This study followed the Consolidated Standards of Reporting Trials (CONSORT) reporting guideline. The trial protocol appears in Supplement 1.

### Participants

A total of 129 patients referred with presumptive knee OA and candidacy for primary TKR were recruited between March 2019 and January 2020. Study subjects were identified during the preclinic team meeting (ie, team huddle). Adult patients aged between 45 and 89 years, fluent in English or Spanish, with body mass index (BMI; calculated as weight in kilograms divided by height in meters squared) between 20 and 46, a primary diagnosis of advanced knee OA (radiographic Kellgren-Lawrence [KL] grade 3 or 4 where grade 0 represents no OA, grade 4 severe OA), baseline Knee Injury and Osteoarthritis Outcome Score for Joint Replacement (KOOS JR) between 0 and 85, able to provide informed consent, and medically fit for TKR were included. Age, BMI, and KOOS JR limits were set based on the estimation model. Exclusion criteria disqualified patients with knee problems not primarily related to OA (eg, trauma, inflammatory arthropathy), advanced OA affecting 1 or both knees with need for care of a different joint problem, prior OA management by an orthopedic specialist, and/or any prior lower extremity total joint replacement.

### Intervention

**Figure 1.**
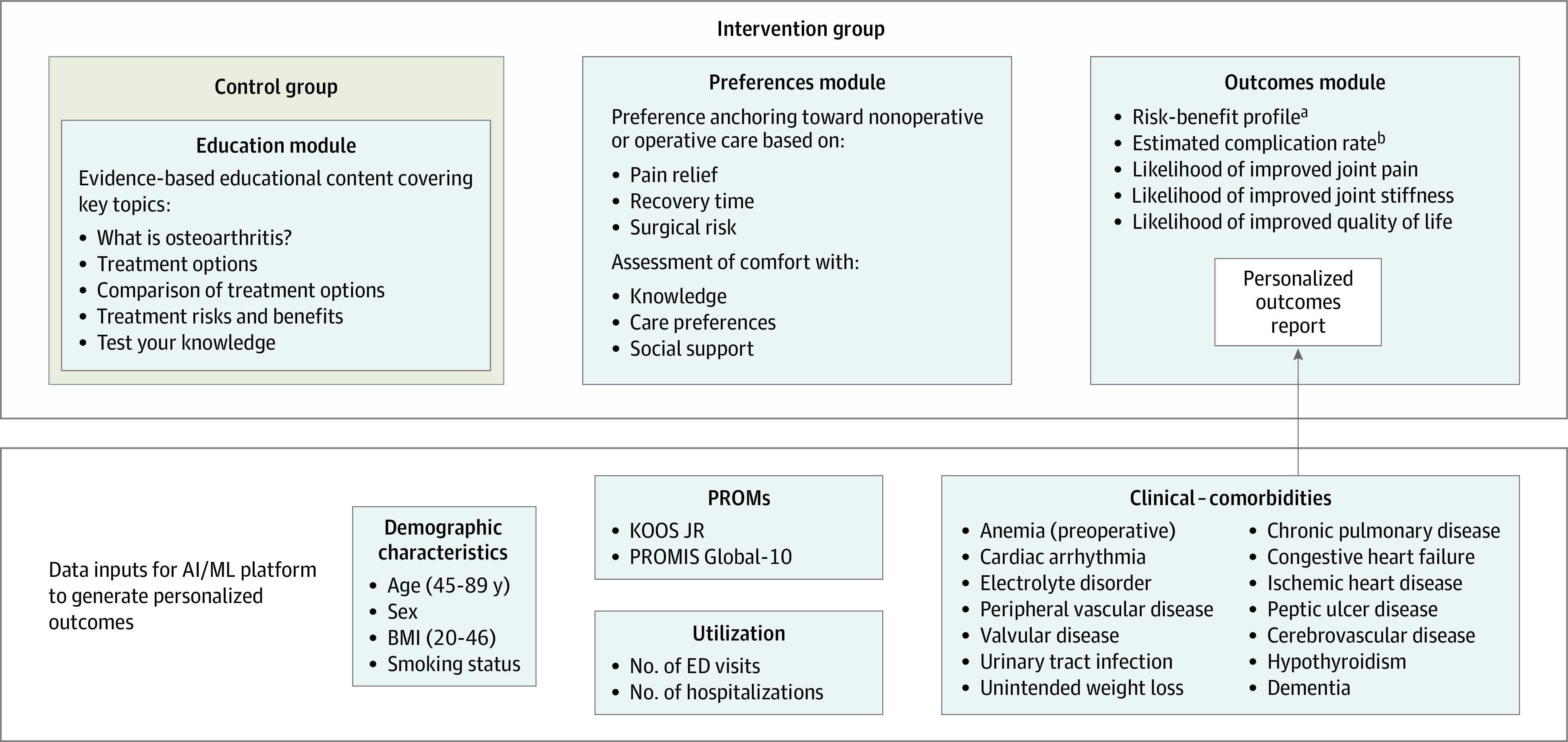
Schematic of Components of Decision Aid and Data Inputs for Artificial Intelligence/Machine Learning (AI/ML) Platform to Generate Personalized Outcomes Schematic visualizing separate components (modules) of the decision aid provided to control and intervention group patients, including data inputs required for the AI/ML platform to generate the personalized outcomes report. BMI indicates body mass index (calculated as weight in kilograms divided by height in meters squared); ED, emergency department; KOOS JR, Knee Injury and Osteoarthritis Outcome Score for Joint Replacement; PROMIS Global-10, Patient Reported Outcome Measurement Instrumentation System Global 10. ^a^Benefit defined as likelihood of experiencing at least a minimal clinically important difference in functional outcome; risk defined as the likelihood of experiencing no change in condition or being worse off after undergoing surgery. ^b^Complication rate defined as estimated complication rate due to joint infection within 90 days, pulmonary embolism or death within 30 days, and pneumonia, sepsis, or acute myocardial infarction within 7 days. Likelihood of improvement in stiffness, pain, and quality of life based on KOOS JR metrics.

The intervention was an AI-enabled patient decision aid incorporating 3 modules (ie, education, preferences, and personalized outcomes) within a software platform (Figure 1). The education module incorporated an overview of the natural history of OA, evidence-based nationally recognized treatment recommendations,^6,22^ comparisons of treatment alternatives, risks and benefits, and a knowledge test developed using guidance from the surgeon authors (K.J.B., J.A., P.J.). The preferences module included ratings for desired levels of pain relief, commitment to postoperative recovery, and willingness to accept surgical risk on a continuum of nonoperative to operative care. Patients also rated whether they gained sufficient knowledge, their self-awareness of their preferences, and their confidence with the level of support received during decision-making. The outcomes module included a personalized report incorporating estimated probabilities of benefits, risks (complications), and likelihood of improvement in joint pain, stiffness, and QoL following TKR, alongside a summary of preferences and education modules. All content was available in English or Spanish, and the decision aid was deployed after a period of familiarization, testing within the clinical setting, and discussions about its fidelity between the clinical team and the company.

### Outcomes

**Figure 2.**
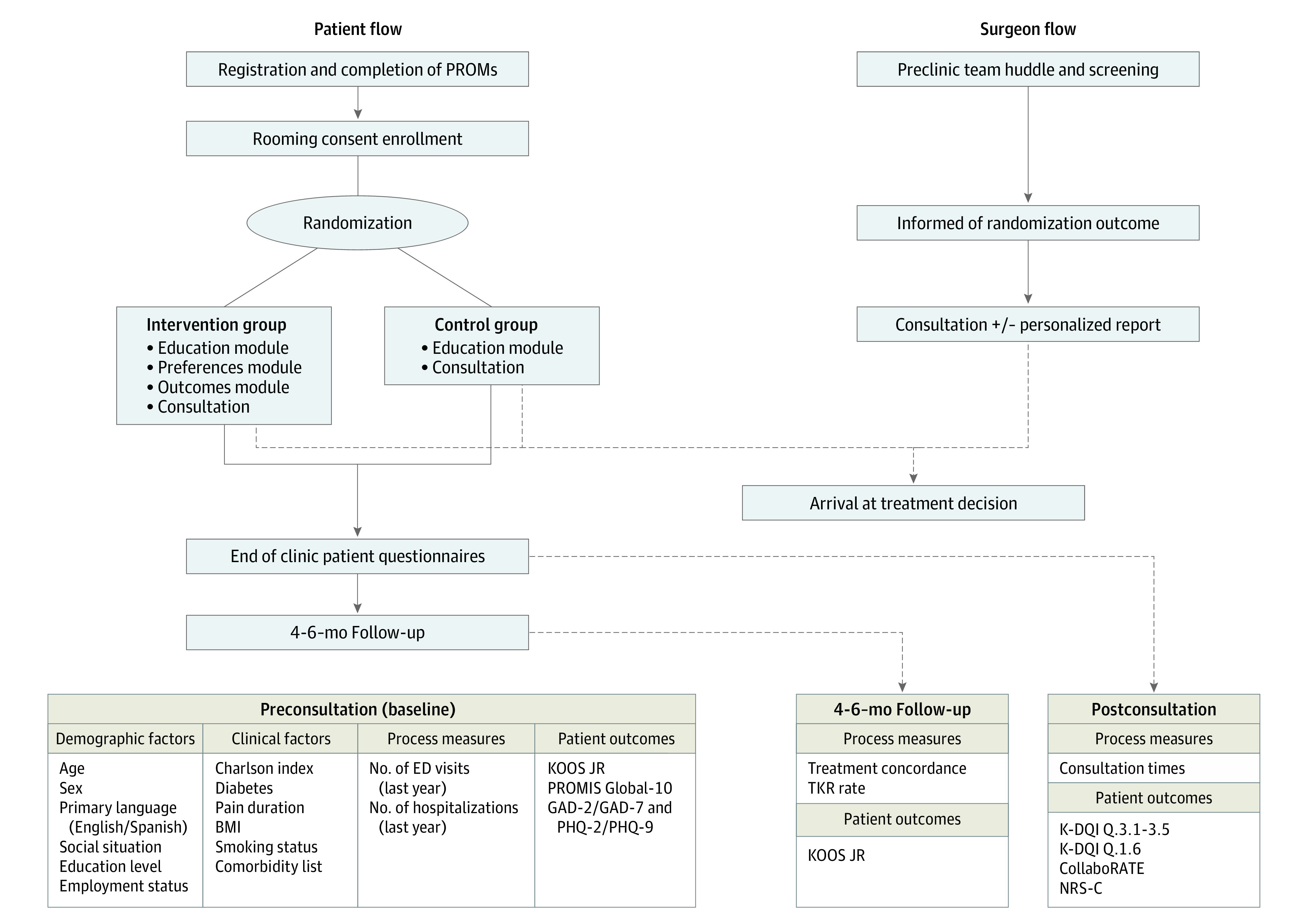
Schematic of Patient Flow and Surgeon Flow Including Demographic, Clinical, and Process Factors and Outcome Measurements at Preconsultation, Postconsultation, and 4-to-6–Month Follow-up Schematic visualizing a step-by-step patient flow and the surgeon flow throughout the study, the timepoints at which demographic, clinical, process, and patient outcome measures are collected. Abbreviations: BMI, body mass index (calculated as weight in kilograms divided by height in meters squared); ED, emergency department; GAD-2/GAD-7, Generalized Anxiety Disorder Questionnaires; K-DQI, Knee Decision Quality Instrument; KOOS JR, Knee Injury and Osteoarthritis Outcomes Survey Joint Replacement; NRS-C, Numerical Rating Scale for Satisfaction; PHQ-2/PHQ-9, Patient Health Questionnaires; PROMIS Global-10, Patient Reported Outcome Measurement Information System Global-10; TKR, total knee replacement.

The primary outcome was the decision process score of the knee decision quality instrument (K-DQI) questions 3.1 through 3.5 (eFigure in Supplement 2) (Figure 2).^24^ Secondary outcomes included the level of SDM (assessed using the CollaboRATE survey),^25^ patient satisfaction with the consultation (numerical rating scale [NRS]),^26^ condition-specific symptoms and functional limitations (KOOS JR),^27^ duration of consultation in minutes, TKR rates (proportion of patients undergoing surgery), and treatment concordance (K-DQI question 1.6) (eFigure in Supplement 2).^24^ All outcomes were assessed at the end of the clinical visit except KOOS JR and treatment concordance, which were assessed prior to the consultation and again at a follow-up appointment 4 to 6 months from initial consultation or date of TKR, as applicable. No changes to trial outcomes were made after study commencement.

### Procedures

All patients completed baseline PROMs after registration that included KOOS JR assessment,^27^ a Patient Reported Outcome Measurement Instrumentation System (PROMIS) Global-10 questionnaire,^28^ Generalized Anxiety Disorder screener (GAD-2)^29^ and full measure (GAD-7)^30^ as indicated, and the Patient Health Questionnaire screener (PHQ-2)^31^ and full measure (PHQ-9)^32^ as indicated. After escort to the clinic room, patients met the research assistant who provided study information and obtained verbal informed consent. We randomized eligible patients to the intervention group or control group using the Randomization Module in REDCap (Research Electronic Data Capture), which also housed our study data.^33^ Demographic characteristics and ethnicity were captured from electronic health records. Ethnicity was classified by patients during clinic registration with preset options; consideration of patient race/ethnicity was included to ensure the study population reflected our patient demographic characteristics and represented minority populations.

Stratified block randomization was used to allot equal numbers of patients from each surgeon’s clinic to each treatment group. An independent project manager performed the random allocation sequence setup with guidance from institutional biostatisticians. Group assignment was revealed after consent and enrollment to research assistants, participants, and surgeons ahead of the consultation. Following randomization, all patients completed a baseline study survey capturing demographic characteristics, clinical factors, and process measures.

The control group received the education module and usual care while those randomized to the intervention group received the education and preferences module before receiving the report from the outcomes module (Figure 2). Patients reviewed the decision aid modules independently prior to consultations following a brief introduction by the researcher who periodically checked in with the patient and remained available to answer any questions. Patients received the personalized report at the same time as the surgeon. Both parties had a chance to review the report prior to the consultation, in which surgeons walked patients through the metrics as part of the discussion. We recorded consultation time by stopwatch, marking surgeon entry and exit from the patient room for the decision-making discussion. Patients completed a final set of questionnaires (K-DQI, CollaboRATE, and NRS) before leaving the clinic, and KOOS JR and treatment concordance were completed at the 4-to-6–month follow-up appointment.

### Statistical Analysis

All primary and secondary outcome measures were examined for distributional properties; data were analyzed from April to May 2020 using Stata statistical software version 16 (StataCorp). Measures from the K-DQI, KOOS JR, and consultation duration were treated as continuous outcomes; NRS and total CollaboRATE scores as ordinal outcomes; and treatment concordance and TKR surgery as binary measures. In testing for intervention effectiveness, assumptions of unequal variance across both groups were checked for continuous measures and, if violated, Satterthwaite adjustments were reported for independent sample *t* tests (ie, K-DQI, consultation time). We fitted linear mixed effect models with random intercepts at the subject level to test the interaction of group assignment with time for KOOS JR. We conducted Mann-Whitney U tests to assess differences between groups for ordinal measures (ie, CollaboRATE, NRS) and performed Fisher exact tests to evaluate differing surgical rates and treatment concordance. To control for inflations of the type I errors rate, we set α = .05 for the primary outcome measures in 2-tailed tests and did the same for secondary outcome measures. The Hochberg-Y procedure was applied to correct individual test α levels in both groups of outcomes to maintain a familywise type I error rate of 0.05 for each.^34^

Power analysis indicated that 130 patients would yield 99% power to detect a comprehensive set of minimum meaningful group differences in K-DQI (15%) and CollaboRATE total scale (2 points), with 63% and 90% power to detect a 7-point or 9-point pre- to postintervention difference in KOOS JR scores, respectively, with α = .017 under a Bonferroni correction (0.05/3). These 3 measures were considered the most directly clinically relevant measures to SDM, decision aids, and patient outcomes in our study.

## Results

Sixty-nine intervention group patients (46 [67%] women) and 60 control group patients (37 [62%] women) were included in the final analysis. Table 1 provides descriptive statistics and counts for demographic and clinical characteristics.

**Table 1.** Descriptive Statistics of Patient Demographic and Other Characteristics by Study Group

Measures	Participants, No. (%)	
	Intervention (n = 69)	Control (n = 60)
Age, mean (SD), y	62.59 (8.85)	62.62 (7.81)
Women	46 (67)	37 (62)
Men	23 (33)	23 (38)
Ethnicity		
White	28 (41)	19 (32)
Asian	7 (10)	9 (15)
Black or African American	11 (16)	11 (18)
Hispanic or Latino	23 (33)	21 (35)
Education		
High school or less	49 (71)	40 (67)
College	10 (14)	16 (27)
Advanced degree	10 (14)	4 (6)
Work status		
Unemployed	23 (33)	23 (38)
Receiving disability	8 (12)	5 (9)
Employed	24 (35)	14 (23)
Retired	14 (20)	18 (30)
Social status		
Living alone	18 (26)	15 (25)
Partnered	49 (71)	41 (68)
Part-time care^a^	2 (3)	4 (7)
Insurance status		
CCC	27 (39)	35 (58)
Commercial	25 (36)	9 (15)
Medicare	16 (23)	16 (27
Self-pay	1 (2)	0
PHQ–depression, mean (SD)	1.9 (2.2)	1.8 (1.9)
GAD–anxiety, mean (SD)	1.2 (0.4)	1.3 (0.5)
Diabetes	11 (16)	16 (27)
Smoking status		
Daily or occasional	8 (12)	7 (13)
Former	18 (26)	11 (18)
Never	43 (62)	41 (69)
Charlson Comorbidity Index, mean (SD)	3.0 (1.6)	2.9 (1.6)
Duration of pain, mean (SD), mo	77.61 (81.96)	70.33 (87.38)
BMI, mean (SD)	32.33 (6.58)	34.27 (6.34)

Abbreviations: BMI, body mass index (calculated as weight in kilograms divided by height in meters squared); CCC, community care collaborative insurance for low-income or underinsured populations; GAD, Generalized Anxiety Disorder; PHQ, Patient Health Questionnaire.

^a^ Part-time care signifies patients live alone but occasionally have some form of home help.

### Participant Flow

**Figure 3.**
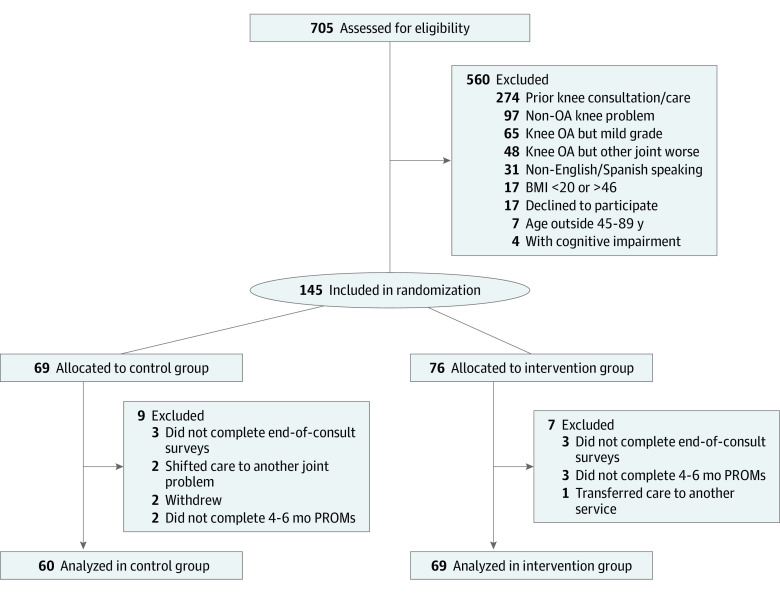
Study Flow Diagram Abbreviations: BMI indicates body mass index (calculated as weight in kilograms divided by height in meters squared); OA, osteoarthritis; PROMs, patient-reported outcome measurements.

During the study period (March 2019 to July 2020, including follow-up), 705 new patients with joint pain presented to the IPU. A total of 560 patients (79.4%) were excluded on initial screening because of exclusion criteria (543 patients [77.0%]) or declining participation (17 patients [2.4%]) (Figure 3). The remaining 145 (20.6%) were randomized into the control group (69 patients) or intervention group (76 patients). In the control group, 9 patients were excluded because of failure to complete postconsultation surveys or follow-up KOOS JR assessment, shifting care to hip or low back pain treatment, or study withdrawal. In the intervention group, 7 patients were excluded after failing to complete postconsultation surveys, transfer of care to another service, or not completing follow-up KOOS JR assessment. There were no harms or unintended effects in either group.

### Outcomes and Estimation

Table 2 provides descriptive statistics and counts for primary and secondary outcome measures. Patients in the intervention group showed better decisional quality (K-DQI, mean difference, 20.0%; SE, 3.0; 95% CI, 14.2%-26.1%; *P* < .0001). Ordinal ratings for level of SDM and satisfaction were highly skewed and consistent with improved outcomes in the intervention group using nonparametric tests. More control patients had scores lower than the CollaboRATE median (also the maximum on the measure) than in the intervention group (28 of 60 [47%] vs 8 of 69 [12%]; *P* < .001). Similarly, 19 of 58 patients (33%) had scores lower than the median value of 10 (also the maximum on the measure) for consultation satisfaction in the control group while 9 of 65 (14%) had scores lower than the median in the intervention group (*P* = .01). Greater improvement in functional outcomes (KOOS JR) was shown from baseline to 4 to 6 months at follow-up (mean [SE], 4.9 [2.1] points higher in intervention group than control group; 95% CI, 0.8-9.0 points; *P* = .02). The intervention group did not experience longer consultation times (mean difference, 2.23 minutes; SE, 2.18). Finally, differential rates of TKR and differential treatment concordance did not reach statistical significance.

**Table 2.** Descriptive Statistics for Primary and Secondary Outcome Measures

Measures	Intervention		Control		*P* value
	Mean (SD)	Participants, No.	Mean (SD)	Participants, No.	
K-DQI	68.9 (19.8)	69	48.8 (14.5)	60	<.001
CollaboRATE, median (range)	27 (22-27)	69	27 (13-27)	60	<.001
Below maximum, %	12	8	47	28	NA
Satisfaction, median (range)	10 (5-11)	65	10 (0-10)	58	<.008
Below maximum, %	14	9	33	19	NA
KOOS JR at baseline^a^	38.8 (16.8)	69	35.7 (19.1)	60	.02
At 4-6 mo	50.7 (18.1)	67	42.8 (19.5)	60	NA
Consultation minutes	27.1 (12.5)	69	24.9 (11.6)	57	.31
TKR treatment, count, No. (%)	16 (23)	69	7 (12)	60	.11
Concordance, count, No. (%)	58 (76)	69	44 (64)	60	.19

Abbreviations: K-DQI, Knee Osteoarthritis Decision Quality Instrument; KOOS JR, Knee Injury and Osteoarthritis Outcome Score Joint Replacement; NA, not applicable; TKR, total knee replacement.

^a^ *P* value is for the interaction effect of time by group in KOOS JR measure from the mixed-effect model.

To assess whether greater TKR rates in the intervention group relative to control group accounted for greater improvement in KOOS JR during the follow-up (although already shown not to be statistically significant), we adjusted for TKR effect in the linear mixed effects model. The relative improvement in KOOS JR remained statistically significant for those in the intervention group (mean [SE], 6.42 [2.31] points higher in intervention group than control group; 95% CI, 1.8-10.9 points; *P* = .02).

## Discussion

TKR—among the most common surgical procedures in the US—is a high stakes encounter for advanced knee OA demanding careful selection.^35,36,37^ The promise of PROMs to improve SDM through enhanced patient engagement has been recognized.^15,38,39^ We developed an AI-enabled decision aid incorporating PROMs, patient education, preference assessment, and personalized estimations of clinical outcomes that improved decision quality, SDM, patient satisfaction, and functional outcomes compared with education and usual care.

Our study joins others that have demonstrated improvements in decision quality, levels of SDM, and greater patient satisfaction in care for knee OA when using decision aids.^41,42,43,44^ Our decision aid produced positive outcomes for individuals from a range of backgrounds, including those experiencing unemployment and those with limited resources. Thus, populations that have been shown to receive less support in making informed care decisions^45,46,47^ may also benefit from this tool. Notably, the relatively low number of patients declining participation indicates that diverse patient populations, including the underserved, also want to participate in their health decisions. Furthermore, both groups in this study had similar characteristics, although commercial insurance was slightly more common in the intervention group despite randomization. For all individuals with advanced knee OA, this decision aid may better address patient expectations around symptom relief, improvement in physical function, and psychosocial wellness, as well as provide clarity around fears, attitudes, and the risks and benefits of surgery.^40,45,48,49,50,51^ Expectation management, patient engagement, and patient-perceived control over decisions synergistically yield optimal outcomes and experiences for those with OA.^52,53,54^ Decision aids addressing patient preferences may equip teams of health professionals to align with these expectations.^55^ Further, the ability of patients to appreciate multiple treatment options and the dynamic nature of their condition is supported by visual elements of our decision aid, including scales for preferences and quantified variability in outcomes based on modifiable personal and clinical factors. Patients using the tool may feel more involved, informed, and in control of the decision-making process; this warrants further study.^56,57^

Notably, few decision aids have demonstrated the positive impact on functional outcomes in knee OA.^15,58^ The findings in our study could be explained by greater patient engagement in those experiencing the full decision aid, which promoted improvements in their ability to perform physical activities. Improvement in PROMs is paramount as payers and policy makers aim to shift toward using patient-centered metrics in value-based payment reforms.^59^ Notably, mental health affects PROMs, with preoperative psychological distress affecting pain, function, and QoL following TKR.^60^ PROMIS Global mental health scores were incorporated in the algorithm, and raw scores were made available for enriching the surgeon-patient discussion.

The combination of PROMs with AI analytics for decision support has also been observed in a 2020 study^39^ that demonstrated the feasibility of a web-based tool providing an estimated outcomes report using previsit PROMs plus clinical risk factors to facilitate SDM for patients with hip and knee OA. While large-scale implementation of this decision aid was achieved, inconsistencies remained around whether the report was reviewed during the decision-making consultation—in contrast to our trial, in which review by both patient and surgeon was mandatory in the intervention group. In another study,^61^ machine learning was shown to estimate postoperative PROMs using preoperative visual analogue scores for pain, Q scores, and clinical factors.

Other studies have shown surgeons experience more efficient use of their time with shorter consultations using decision aids.^41^ Our study found no significant change in consultation time in either direction despite the inclusion of a joint review of the personalized outcomes report during the consultation.

In relation to TKR use, some authors suggest that informed patients opt for more conservative treatments.^62,63^ Indeed, reduced surgical rates have been demonstrated.^42,64,65^ However, we advise caution in relying on these tools to steer patients toward less invasive treatment options; our previous work exhibited no differential impact on TKR use between decision aid users and nonusers.^41^ Interestingly, while outside our target population, those with less severe OA may also benefit from experiencing similar analytics, given that in these cases decision aids are likely to reveal (and underline) less favorable outcomes from undergoing surgery. Advanced decision support may lead patients toward evidence-based nonoperative strategies and surgeons toward safe and judicious use of TKR. This may be a powerful asset as volume and expenditure for TKR increase.^11,66,67,68,69,70,71^ More informed patients with more realistic preoperative expectations are less likely to be dissatisfied with the results of their operations, and therefore may use fewer resources (eg, extended physical therapy or pain management) postoperatively.^48,72^

Notably, the lack of significant impact on treatment concordance between our study groups may be explained by the standard of care in the IPU, where set treatment plans during initial consultations are usually followed through (ie, treatment performed matches treatment selected) irrespective of decision aids more focused on the decision-making process itself than the outcome of the decision. Lastly, the decision aid in this study incorporates complex analytics that require careful and clear explanations to patients. While no formal training was provided to surgeons, future implementation of such tools should incorporate coaching for clinicians to put the data generated in human terms and effectively communicate insights as treatment options. Decision aids have incorporated communication aids to enhance the health care professional–patient interaction,^41^ and this tactic could be applied to our tool. Widespread uptake of such innovations will ultimately depend on surgeons attesting to data insights supporting their deductive reasoning and judgment during SDM, data flow automation (to minimize user burden), robust longitudinal PROM collection, coaching on SDM methods, and communication of outputs alongside legal and ethical considerations.^59,64,65,73,74^ Addressing these factors may accelerate the successful integration of AI-enabled decision aids using PROMs and drive uptake of these tools to unlock advanced insights as the machine learns from an accumulating set of clinical- and patient-focused data points to provide reliable, real-time estimations of outcomes.

### Limitations

There are several limitations to this study. First, this work was performed in a specialized setting at a single institution delivering a comprehensive range of treatments. While this potentially limits generalizability, the study does demonstrate the feasibility of using this type of decision aid in any setting where PROMs are collected longitudinally. Future evaluations should account for nonoperative strategies delivered and assess outcomes in different settings, including traditional fee-for-service care. Second, because surgeons were not masked to the intervention, there is potential for contamination (ie, differential surgeon behavior expressed toward each group, such as motivational bias manifest in enhanced interactions with those in the intervention group). Such biases were challenging to accommodate in our study design. Third, we did not assess the effect of the decision aid on patient knowledge, because matching educational content was provided to both groups nor did we assess patient activation (defined as propensity to engage in adaptive health behaviors), choice awareness, aspects of deliberation (eg, decision conflict and decision regret), expectation management, or surgeon perceptions (eg, efficiency of the consultation). While research assistants were available to answer any questions patients had while they read the education material and used the decision aid, we did not formally assess health literacy, and some patients may not have been able to read or understand all content in the decision aid. Future iterations of the decision aid may also include audio or video options instead of having to read the material. Further work should explore these factors while minimizing questionnaire burden and account for health literacy, language barriers, and sociodemographic status.^40^ Fourth, the typical course of a formal OA in-clinic diagnosis poses a general limitation in limiting the timeframe over which the tool may be applied.

## Conclusions

The findings of this study suggest that multifaceted decision aids integrating patient education, preference assessment, and AI-enabled analytics built with PROM data can provide a personalized, data-driven approach to SDM for patients with advanced knee OA considering TKR. Benefits to patients observed in this single-center study warrant further investigation across multiple sites routinely collecting PROMs, with careful consideration of institutional and health care professionals’ experiences of incorporating AI in practice. The patient-centered, data-driven approach to SDM in this study may mark a step-change in the application of patient decision aids in orthopedic practice.

## Notes

Supplement 1. **Trial Protocol**

Supplement 2. **eFigure.** Knee-Decision Quality Index (Decision Quality Component and Concordance Component)

Supplement 3. **Data Sharing Statement**
