# Supplementary material for: Comparison of an Artificial Intelligence–Enabled Patient Decision Aid vs Educational Material on Decision Quality, Shared Decision-Making, Patient Experience, and Functional Outcomes in Adults With Knee Osteoarthritis: A Randomized Clinical Trial
Source: JAMA Netw Open. 2021 Feb 18;4(2):e2037107. doi: 10.1001/jamanetworkopen.2020.37107 (PMC7893500; doi:10.1001/jamanetworkopen.2020.37107)
Supplement: Supplement 1. — Trial Protocol [file jamanetwopen-e2037107-s001.pdf]

PROTOCOL

Impact of an Artificial Intelligence-Enabled Decision Aid on Decision Quality, Shared Decision Making, Patient Experience and Functional Outcomes in the Management of Knee Osteoarthritis: A Randomized Clinical Trial

Objectives

Assessing impact of a technology-enabled decision aid on:

- Knee decision quality	**K-DQI survey**
- Shared decision-making	**CollaboRATE survey**
- Patient experience (satisfaction with decision-making process	**NRS Satisfaction**
- Change in functional outcomes	**KOOS JR**
- Processes	**Consult duration / TKR Rate**
- Concordance	**Alignment of treatment choice**

Subject Selection

Inclusion Criteria (to be checked during/after huddle/after x-ray/at consent):

i) All new patients with a presumptive diagnosis of knee OA aged between 45 and 89 years*
ii) Patients fluent in English or Spanish
iii) BMI between 20 and 46*
iv) Primary diagnosis of advanced knee OA (K-L grade of 3 or 4 i.e. moderate to severe)
v) KOOS JR scores between 0-85*
vi) Participant able to give informed consent for participation in the study
vii) Medical fitness for TKR

* Age, BMI, KOOS JR cut-offs based on range limits set by decision aid algorithm

Exclusion Criteria:

i) Patients seeking care for knee problem not primarily related to knee OA e.g. trauma condition, or psoriatic/rheumatoid arthritis
ii) Patient with advanced knee OA but primarily requiring care for a different joint problem
iii) Patients with a prior experience of lower extremity total joint replacement or OA work-up
iv) Prior experience with the pilot form of this decision aid tool
v) Patients undergoing consideration for revision joint replacement

1. **Subject Recruitment**

Sources

Subjects will be identified and recruited through the Lower Extremity Musculoskeletal Institute (MSKI) Integrated Practice Unit (IPU) Clinic schedule at the University of Texas at Austin / Dell Medical School. The subjects will be identified as new patients presenting with knee pain with a presumptive diagnosis of OA and a scheduled appointment with Dr. Kevin Bozic or Dr. Karl Koenig, the two lead orthopaedic surgeons for respective clinics. Patients will be randomized to the AI-enabled decision aid (Intervention group, Group A), or usual care with patient education only (Control group, Group B).

Recruitment methods

Patients will be identified as suitable candidates for the study during the pre-clinic meeting (huddle). Once the patient has entered the clinic room, they will be met by a researcher and invited to participate in the study then provide informed consent. If they agree to partake, the researcher will perform randomization using an online platform (see T0.2 Randomization) inside or outside the clinic room depending on internet access. Patients will be informed if they are in the intervention group (full decision aid i.e. education, preference and personalized outcomes modules, denoted Gp A) or control group (educational module only and usual care, denoted Gp B). Researchers will be provided with standard script for explanations (see below in relevant sections).

2. Enrollment / Consent Process and Documentation

Enrollment

- All potential patients assessed for eligibility will be entered into an Excel worksheet.
- Documentation will include:
    - Enrollment Status:
        - Consented (patients consented to be part of the study and randomized into either intervention or control group)
        - Declined:
            - Reason for decline:
                - Not enough time
                - Privacy concerns
                - Not interested
                - No reason given
                - Other (record reason)
        - Screening failure:
            - Reason for screening failure
                - Not OA
                - Patient had a prior experience of TJA / TJA discussion
                - Patient was being considered for revision joint replacement
                - Patient seeking care for trauma condition or other joint concern
                - Non-English or Non-Spanish speakers
                - BMI below 20 or above 46
                - Age under 45 or over 89
                - Unable to give informed consent / issue with cognitive status
                - Other
        - Withdrawn by PI
                - Etiology of joint pain is not OA
                - Patient had a prior experience of TJA
                - Patient had a prior experience of the decision aid
                - Patient was being considered for revision joint replacement
                - Patient seeking care for trauma condition
                - Non-English or Non-Spanish speakers
                - Patients with BMI below 20 or above 46
                - Age under 45 or over 89
                - Participant unable to give informed consent / issue with cognitive status or ability
                - Other

- Withdrawn by patient (patient was enrolled, randomized, and decided not to participate in the study)

- The centralized REDCap will be utilized to keep track of patients deemed not eligible during the morning huddle on each clinic day, and specific rationale for exclusion (i.e. exclusion criteria) will be delineated.
    - Etiology of joint pain is not OA
    - Patient had a prior experience of TJA
    - Patient had a prior experience of the decision aid
    - Patient was being considered for revision joint replacement
    - Patient seeking care for trauma condition or other joint concern
    - Non-English or Non-Spanish speakers
    - Patients with BMI below 20 or above 46
    - Age under 45 or over 89
    - Participant unable to give informed consent / issue with cognitive status
    - Other - specify

Consent Process

A research associate (RA) will explain the purpose of the study, what is involved in the study, the risks and benefits of the study, and determine if the patient is interested in participating – standard prose will be provided to all research assistants (see below in relevant sections). Consenting patients will be randomized into either the intervention (Gp A) or control group (Gp B). At any time, potential subjects can request additional time to think about the study or decline participation. All questions the patient may have will aim to be answered to the best of their ability by the research assistant, project leads or PI. At the time of this initial consultation, the patient will be asked to sign a paper consent form and offered a blank form for their records.

3. <u>Procedures</u>

<u>Intervention Arm</u>

T0. Screening/Study Recruitment

See above.

T0.1 Patient Enrollment

Assess all new patients for eligibility according to the schedule. Patients who are not eligible and therefore will not be approached for enrollment will be noted as such along with the reason for not approaching the patient on a spreadsheet specifying one of the rationales stated above in #2.

"Hi, my name is ______. I'm working with Dr. Bozic / Dr. Koenig, your physician today, on a study involving software that aims to support treatment decisions related to knee arthritis. This tool provides educational materials on your condition—the reason for your knee pain—and uses your age, sex, BMI and questions about health you already answered on the iPads to make personalized calculations of risks, benefits, and aspects related to your knee problem.

The purpose of this study is to learn more about the impact of the software on aspects of decision making and assess your satisfaction with the decision making process. This will be done using questionnaires at

the end of the visit. If you agree to participate, we will let you know whether you were randomly selected to a group receiving educational materials, a risk:benefit report, and some interactive components or a group receiving educational materials only.

This is a fair way of studying an intervention, in this case the software, and learn about its impact. Regardless of what group you are in, after consultation you will be asked to complete a set of questionnaires that should take less than 10 minutes. Questions will be about the quality of decision made, how well your preferences were considered in making the decision, knowledge on knee replacement surgery, and your satisfaction with the decision-making process."

The tool ***does not*** *replace the usual care you receive at our clinic so you will still see all the health care professionals you would usually meet and be able to discuss all the things you want to discuss with each member of our team.*

Your participation is completely voluntary and does not impact whether you will receive care. No penalty will be applied should you decide not to participate. Do you have any questions? Do you agree to take part in the study regardless of the group you may be assigned? Would you like more time to think about it?"

If yes…
"Thank you so much for your agreement to participate. I will now momentarily randomize you into either Group A or Group B. Regardless of which group you are assigned, your continued participation is very important for our study outcomes.

If no…
"That's okay. Could you kindly tell me your reason why? This is useful for feedback and enables us to improve our studies in future..."

T0.2 Randomization

Randomize the patient in the following manner:

- Go to Add/Edit Records on the left hand side of REDCap, then "Add new record"
- Answer whether or not the patient consents to be included.
- If the patient consents, select the clinic/surgeon, then hit the "Randomize" button.
- Inform the patient as to the group into which they have been randomized (see script below).
- Change form status to Complete, then go to top right to "Survey Options" and then hit Submit before handing to patient.

Values/labels for "randomization_group" (Randomization Group):
1 Group A - Full tool
2 Group B - Educational materials only

Values/labels for "clinic" (Site & Clinic-Associated Surgeon):
1 UT Health Austin - Bozic
2 UT Health Austin - Koenig

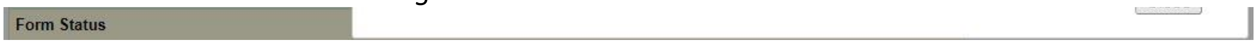

- *For those assigned Group A:*

"You have been selected to receive the educational materials, a calculated risk:benefit report and some interactive components.

"Let me briefly talk you through the process. Please kindly read the education material on this iPad at your own pace, answer the questions at the end, and use the sliders to tell us about your preferences and experiences. You will then receive a report customized to you which can guide discussion with your provider. Please feel free to ask any questions during this consultation. Thanks in advance for completing some survey questions on how it all went at the end of the visit, please be sure to wait for me to return before leaving."

- *For those assigned Group B:*

"You have been selected to receive the educational materials."

"Let me briefly talk you through the process. Please kindly read the education material on this iPad at your own pace. You will then have a chance to discuss you problem and treatment options with your provider. Please feel free to ask any questions during this consultation. Thanks in advance for completing some survey questions on how it all went at the end of the visit, please be sure to wait for me to return before leaving."

For Group A - Both provider and patient will have printed copies of the risk:benefit report, answers to a set of 5 test questions patients are provided following the education information, and the interactive components (e.g. preference scales, sense of knowledge, preferences, and values..) that will be referred to during the consultation. Following this discussion, patients will be asked to complete the K-DQI, CollaboRATE survey, NRS-C before leaving clinic. They will complete the KOOS JR at 6 weeks and 6 months following the visit as per standard of care.

For Group B – Patients will read the educational material and prior to their consultation with their provider. Following this discussion, patients will be asked to complete the K-DQI, CollaboRATE survey, NRS-C before leaving clinic. They will complete the KOOS JR at 6 weeks and 6 months following the visit as per standard of care.

T0.3 Reasons for ineligibility

The patient is ineligible for the study if any of the following comes to light at any point during the clinic visit:

- Patient declined
- Screening failure
- Patient withdrawal
- PI withdrawal of patient
    - Reasons:
        - Patient or provider determination that pt. does not have OA
        - Patient had a prior experience of TJA / TJA discussion
        - Patient was being considered for revision joint replacement
        - Patient seeking care for trauma condition
        - Non-English or Non-Spanish speakers
        - Patients with BMI below 20 or above 46
        - Age under 45 or over 89
        - Participant unable to give informed consent / issue with cognitive status
        - Other

These reasons will be documented with patient informed: "As part of the study it is useful for us to note the reason behind why patients decline to participate. May I ask for your reason?"

T0.4 Patient FAQs

Ask if there are any further questions. Some FAQ from patients are:

- Will I have to make any additional trips for the purposes of this study?
  - No, everything that is not conducted on the day of your appointment will be conducted either over the phone or via email.
- Will this involve any experimental procedures or drugs?
  - No, the only experimental part of this study consists of whether you receive just educational materials or educational materials plus a risk:benefit report.
- How long will this take?
  - The total commitment time for the study will be approximately 1 hour over a period of 6 months.
- How will this affect my appointment?
  - Your appointment will be conducted in the usual manner. This study will not affect the doctor's or medical team's actions.
- Will my information be released to insurance companies?
  - No, your information will remain private.

T1.1: Pre-Consultation

RA will populate the patient profile on the OM1 portal, thereby generating a token for the patient to access the in-browser educational materials on the iPad.

- Responses will be recorded by the OM1 system.
- To fill all fields, RA must question patient about 1) hospitalizations and emergency department visits over the past 12 months, and 2) confirmation of any conditions listed on the health history form located visible in AthenaHealth.
- The RA will also obtain height and weight from the Vitals section of AthenaHealth.

T1.2 PRO population

While patient is reading over the iPad materials and answering questions embedded in these educational materials, the RA completes the patient's OM1 profile utilizing individual patient-reported outcome measure question responses to the KOOS, JR and PROMIS-10 Global as viewable in AthenaHealth.

T1.3 Risk:Benefit Report Generation

RA ensures completion of all details required to generate the customized report, including a second module (for bilateral knee pain) where applicable. RA will print two copies of this report, placing one inside the exam door basket (with verbalization to the clinician) and handing the other to the patient with encouragement to use this as a guide throughout the appointment. RA reminds the patient that he/she will return following clinician consultation.

T1.4 Timing Prep

Research assistant will remind clinician seeing any enrolled patient to write with dry erase marker on the status whiteboard to indicate time entering and time leaving the exam room.

REDCap timestamps will be used to clock the duration of the visit, since both pre-surveys and post-surveys will be administered.

T2: Post-Consultation

T.2.1. Patient Post-Consultation

After the patient's consultation, he/she will be asked to complete a post-consultation survey, stage of decision making, and visit satisfaction survey. These surveys will be administered by the RA.

Retrieve the iPad from the patient once they are finished and check for completeness.

T.2.2. Research Assistant Process Post-Consultation

RA will fill in the "Initial Visit Details" form on REDCap, utilizing patient details from the AthenaHealth EMR for patient-reported outcome scores and comorbidities specified. This form includes a question on duration of time provider spent with patient – this will be calculated via subtraction of the entry time from the exit time by the research assistant based on the entry/exit times written on the status board by the provider. In cases where timings were missed, the researcher will confirm duration estimates (in minutes) with the provider.

The RA will also communicate with the provider(s) who saw the patient regarding the patient's final decision on surgery or no surgical intervention.

Timeline of data collection: Majority of the measures are acquired at the baseline consultation visit, while a few are extracted from electronic health record (e.g. actual treatment after consultation). Physical/ functional limitations on KOOS JR, however, are acquired at both the baseline consultation visit and at follow-up appointment 4-6 months out from initial consultation.

T3: 6 Months Post

Patients will be called by study personnel to complete a KOOS JR survey via telephone or emailed link in the case that a new (6-month follow-up) KOOS JR score has not already been obtained by the UTHA call team; as per standard of care, there is a 6-month call, so calls by the investigator team will be made only in the case that these scores are not successfully recorded by usual UTHA outreach.

Patients Opting out of Participation

Patients who do not wish to participate in the study will continue with their normal course of medical care. They will be marked as "Declined."

Patients Withdrawn from the Study by the physician

The patient may be withdrawn from the study following the consultation if the physician deems that the patient meets one or more of the exclusion criteria. The status for these patients will be changed to "Withdrawn."

4. Statistical Analysis

Primary outcome measures:

- Decision quality using percent scaling from the Decision Quality Index (DQI),

Secondary outcome measures:

- Sense of collaboration in decision making total scale score obtained from CollaboRATE
- Physical/functional limitations scale from the KOOS JR
- Duration of initial consultation
- Satisfaction with consultation from the NRS
- Actual treatment outcome as either TKA or non-surgical options
- Concordance between initial treatment preference and actual treatment outcome.

Statistical Analysis Plan

Measures from the DQI, KOOS JR, and consultation duration will be treated as continuous. Assumptions of unequal variance across the two groups will be checked for these continuous measures and if violated, Satterthwaite adjustments will be reported for independent sample t-tests.

Satisfaction, collaboration total scores were treated will be treated as ordinal. Mann-Whitney U tests were conducted to test differences between these groups.

Treatment outcome and concordance will be treated as binary measures.

To accommodate all available information in the context of missing data points, linear mixed effect models with random intercepts at the subject level can be fitted to test the interaction of group assignment with time.

We will use Fisher's exact test to evaluate whether the two study arms differed in total knee replacement surgery rates and concordance between initial treatment preference and eventual treatment outcome.

To control for inflations of type-I errors rate, we will set alpha = .05 for the group of primary outcome measures and did the same for the group of secondary outcome measures. The Hochberg-Y procedure was applied to correct individual test alpha's in both groups of outcomes to maintain a familywise Type I error rate of .05 for each (Hochberg, 1988).

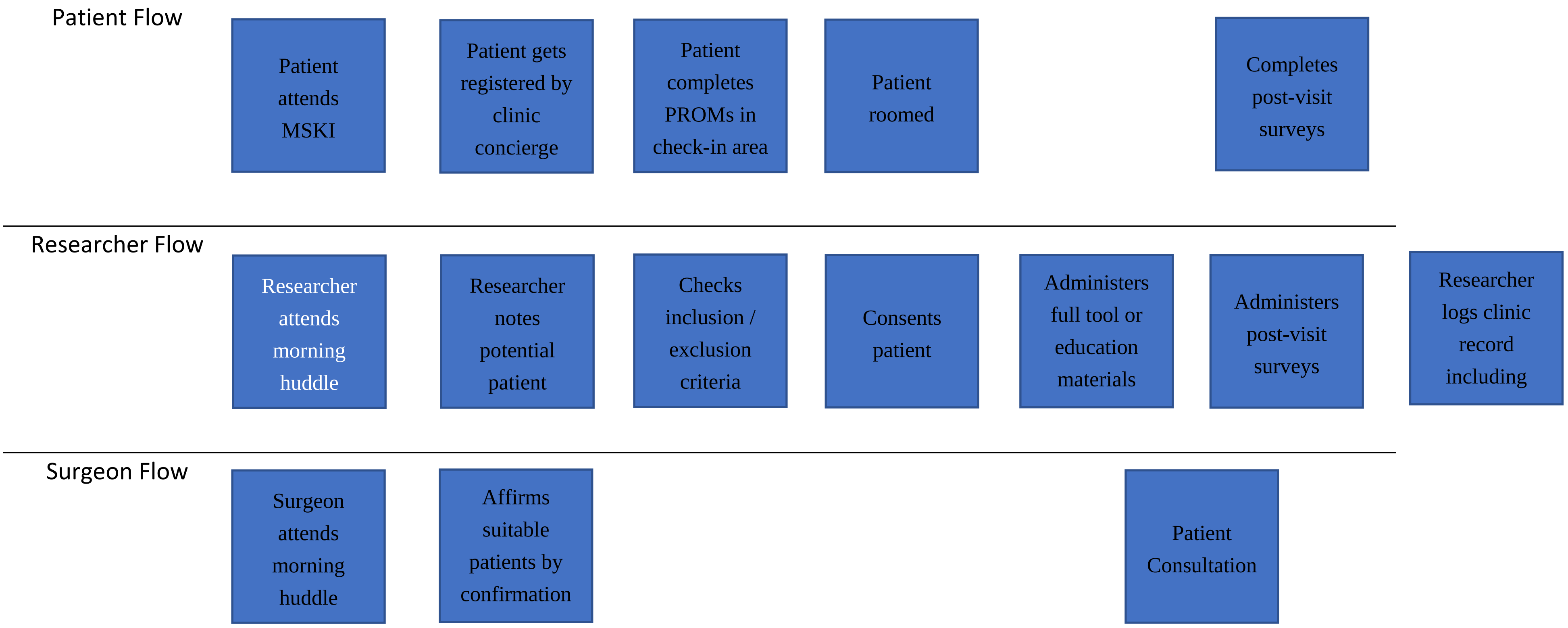
Patient Flow
Patient attends MSKI
Patient gets registered by clinic concierge
Patient completes PROMs in check-in area
Patient roomed
Completes post-visit surveys
Researcher Flow
Researcher attends morning huddle
Researcher notes potential patient
Checks inclusion / exclusion criteria
Consents patient
Administers full tool or education materials
Administers post-visit surveys
Researcher logs clinic record including
Surgeon Flow
Surgeon attends morning huddle
Affirms suitable patients by confirmation
Patient Consultation
