# Supplementary material for: Comparison of an Artificial Intelligence–Enabled Patient Decision Aid vs Educational Material on Decision Quality, Shared Decision-Making, Patient Experience, and Functional Outcomes in Adults With Knee Osteoarthritis: A Randomized Clinical Trial
Source: JAMA Netw Open. 2021 Feb 18;4(2):e2037107. doi: 10.1001/jamanetworkopen.2020.37107 (PMC7893500; doi:10.1001/jamanetworkopen.2020.37107)
Supplement: Supplement 2. — eFigure. Knee-Decision Quality Index (Decision Quality Component and Concordance Component) [file jamanetwopen-e2037107-s002.pdf]

Supplemental Online Content

Jayakumar P, Moore MG, Furlough KA, et al. Comparison of an artificial intelligence–enabled patient decision aid vs educational material on decision quality, shared decision-making, patient experience, and functional outcomes in adults with knee osteoarthritis: a randomized clinical trial. *JAMA Netw Open*. 2021;4(2):e2037107. doi:10.1001/jamanetworkopen.2020.37107

eFigure. Knee-Decision Quality Index (Decision Quality Component and Concordance Component)

This supplemental material has been provided by the authors to give readers additional information about their work.

© 2021 Jayakumar P et al. JAMA Network Open.

eFigure. Knee-Decision Quality Index (Decision Quality Component and Concordance Component)

DECISION QUALITY WORKSHEET
TREATMENTS FOR KNEE OSTEOARTHRITIS

Instructions

This survey has questions about what it is like for you to make decisions about treating your knee osteoarthritis.

Section 1: What Matters Most to You

1.6. Which treatment do you want to do to treat your knee osteoarthritis?

- ☐ Knee replacement surgery
- ☐ Non-surgical treatment options
- ☐ I am not sure

Section 3. Talking with Health Care Providers

Please answer these questions about what happened when you talked with health care providers including doctors, nurses and other health care professionals about knee replacement surgery and other non-surgical treatments, such as exercise or medicine, for knee osteoarthritis.

3.1. Did any of your health care providers talk about knee replacement surgery as an option for you?

- ☐ Yes
- ☐ No

3.2. How much did you and your health care providers talk about the reasons to have knee replacement surgery?

- ☐ A lot
- ☐ Some
- ☐ A little
- ☐ Not at all

3.3. How much did you and your health care providers talk about the reasons **not** to have knee replacement surgery?

- ☐ A lot
- ☐ Some
- ☐ A little
- ☐ Not at all

3.4. Did any of your health care providers talk about non-surgical treatments as something that you should seriously consider?

- ☐ Yes
- ☐ No

3.5. Did any of your health care providers ask you whether you wanted to have knee replacement surgery or not?

- ☐ Yes
- ☐ No

© 2021 Jayakumar P et al. JAMA Network Open.
