# Supplementary material for: Comparison of an Artificial Intelligence–Enabled Patient Decision Aid vs Educational Material on Decision Quality, Shared Decision-Making, Patient Experience, and Functional Outcomes in Adults With Knee Osteoarthritis: A Randomized Clinical Trial
Source: JAMA Netw Open. 2021 Feb 18;4(2):e2037107. doi: 10.1001/jamanetworkopen.2020.37107 (PMC7893500; doi:10.1001/jamanetworkopen.2020.37107)
Supplement: Supplement 3. — Data Sharing Statement [file jamanetwopen-e2037107-s003.pdf]

Data Sharing Statement

Data
Data available: Yes
Data types: Deidentified participant data, Data dictionary
How to access data: lauren.uhler@austin.utexas.edu
When available: With publication

Supporting Documents
Document types: Statistical/analytic code, Informed consent form
How to access documents: lauren.uhler@austin.utexas.edu
When available: With publication

Additional Information
Who can access the data: Researchers whose proposed use of the data has been approved
Types of analyses: Only for purposes specified in data request.
Mechanisms of data availability: After approval of a proposal and with a signed data access agreement.

Data Sharing Statement

Individual participant data that underlie the results reported in this article, after deidentification, will be available (including data dictionaries) alongside the study protocol, statistical analysis plan and analytic code beginning 9 months and ending 36 months following article publication. Investigators who provide a methodologically sound proposal and whose proposed use of participant data is approved by our institutional review board for studies, such as a meta-analyses, will be allowed access. Proposals should be directed to lauren.uhler@austin.utexas.edu. To gain access, data requesters will need to sign a data access agreement. Data are available for 36 months following article publication. After 36 months, the data will be available in our Universitie's data warehouse but without investigator support other than metadata.
